# 576. Chandipura virus infection: Time to think beyond Encephalitis?

**DOI:** 10.1093/ofid/ofae631.014

**Published:** 2025-01-29

**Authors:** Kathan P Buch, Pankaj M Buch

**Affiliations:** GCS Medical College, Hospital and research centre, Rajkot, Gujarat, India; PDU Medical College, Rajkot, Gujarat, Rajkot, Gujarat, India

## Abstract

**Background:**

Chandipura virus, first isolated in 1965, has caused several outbreaks in India, including significant ones in 2003-2004 in central India and in 2024 in western India. Typically affecting children aged 6-15, recent cases have shown an increase in extra-neurological manifestations.

Baseline characteristics of patients

**Figure:**
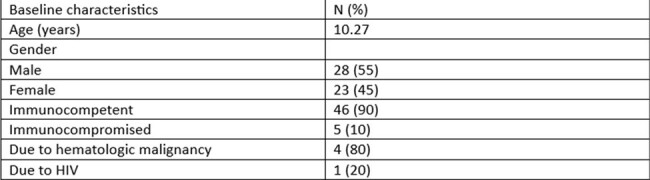

**Methods:**

This study, conducted at a tertiary care hospital in western India from July 1 to August 13, 2024, examined the epidemiology, clinical profile, and treatment outcomes of patients with Chandipura virus infections. It was a single-center, observational study including all patients with confirmed Chandipura virus infection (RT-PCR positive in CSF or sera).

laboratory characteristics of Patients

**Figure:**
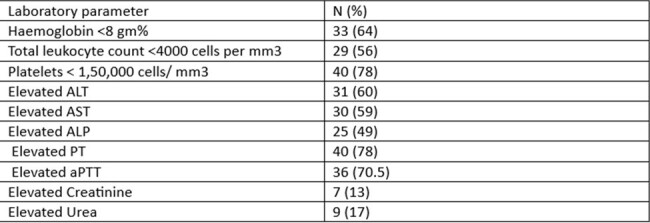

**Results:**

*Baseline Characteristics*: This study involved 51 patients with a mean age of 10.27 (2.32) years, with 55% being male. All the patients were from endemic areas of Gujarat in India. The majority of the patients (90%) were immunocompetent. All cases (100%) were diagnosed positive on RT-PCR of CSF or sera.

Clinical and Laboratory Characteristics:

Common symptoms included fever (100%), headache (70%), altered sensorium (75%), circulatory collapse and shock (35%), and convulsions (25%). Symptoms lasted an average of 5.1 days. Liver dysfunction occurred in 60% of cases, and 19% had acute kidney injury. Abnormal PT, aPTT, and INR levels were found in up to 75% of cases. The main diagnoses were acute viral encephalitis (50%) and encephalitis with MODS due to circulatory collapse (40%).

All 51 patients received standard treatment per Indian government guidelines, including IV fluids, paracetamol, and management of airway, breathing, circulation, intracranial hypertension, and seizures. The median hospital stay was 6 days. Of the 51 patients, 37 died—21 from circulatory collapse and 16 from encephalitic symptoms. The remaining 14 survivors will be monitored for residual neurological issues.

**Conclusion:**

In high-dengue-endemic countries like India, diagnosing CHPV, which can mimic hemorrhagic fever, is challenging. Diagnosis is often delayed, leading to poorer outcomes. Most patients showed symptoms of hemorrhagic fever combined with encephalitic features, highlighting the need for further research on CHPV’s impact on blood vessel endothelial cells.

**Disclosures:**

**All Authors**: No reported disclosures

